# A second polymorph with composition Co_3_(PO_4_)_2_·H_2_O

**DOI:** 10.1107/S1600536808028365

**Published:** 2008-09-13

**Authors:** Young Hoon Lee, Jack K. Clegg, Leonard F. Lindoy, G. Q. Max Lu, Yu-Chul Park, Yang Kim

**Affiliations:** a Department of Chemistry, Kyungpook National University, Daegu, 702-701, Republic of Korea; b Centre for Heavy Metals Research, School of Chemistry, F11, University of Sydney, New South Wales 2006, Australia; c ARC Centre of Excellence for Functional Nanomaterials, AIBN, University of Queensland, Brisbane, Queensland 4072, Australia; d Department of Chemistry and Advanced Materials, Kosin University, 149-1 Dongsam-dong, Yeongdo-gu, Busan 606-701, Republic of Korea

## Abstract

Single crystals of Co_3_(PO_4_)_2_·H_2_O, tricobalt(II) bis­[ortho­phosphate(V)] monohydrate, were obtained under hydro­thermal conditions. The compound is the second polymorph of this composition and is isotypic with its zinc analogue, Zn_3_(PO_4_)_2_·H_2_O. Three independent Co^2+^ cations are bridged by two independent orthophosphate anions. Two of the metal cations exhibit a distorted tetra­hedral coordination while the third exhibits a considerably distorted [5 + 1] octa­hedral coordination environment with one very long Co—O distance of 2.416 (3) Å. The former cations are bonded to four different phosphate anions, and the latter cation is bonded to four anions (one of which is bidentate) and one water mol­ecule, leading to a framework structure. Additional hydrogen bonds of the type O—H⋯O stabilize this arrangement.

## Related literature

Besides crystals of the title compound, crystals of the related phase Co_3_(PO_4_)_2_·4H_2_O (Lee *et al.*, 2008 ▶) were also obtained under hydro­thermal conditions. For a review of metal complexes of organophosphate esters and open-framework metal phosphates, see: Murugavel *et al.* (2008 ▶). For different cobalt(II) phosphates, see: Mellor (1935 ▶). The first polymorph of composition Co_3_(PO_4_)_2_·H_2_O was reported by Anderson *et al.* (1976 ▶), and the crystal structure of the isotypic Zn analogue Zn_3_(PO_4_)_2_·H_2_O was described by Riou *et al.* (1986 ▶).

## Experimental

### 

#### Crystal data

Co_3_(PO_4_)_2_·H_2_O
*M* *_r_* = 384.75
Monoclinic, 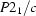

*a* = 8.7038 (15) Å
*b* = 4.8667 (9) Å
*c* = 16.705 (3) Å
β = 95.670 (3)°
*V* = 704.1 (2) Å^3^
*Z* = 4
Mo *K*α radiation
μ = 7.47 mm^−1^
*T* = 150 (2) K
0.46 × 0.14 × 0.08 mm

#### Data collection

Siemens SMART 1000 CCD diffractometer
Absorption correction: multi-scan (*SADABS*; Sheldrick, 1999 ▶) *T*_min_ = 0.247, *T*_max_ = 0.554
6569 measured reflections
1697 independent reflections
1603 reflections with *I* > 2σ(*I*)
*R*_int_ = 0.026

#### Refinement

*R*[*F* ^2^ > 2σ(*F* ^2^)] = 0.033
*wR*(*F* ^2^) = 0.095
*S* = 1.07
1697 reflections
133 parameters
2 restraints
H atoms treated by a mixture of independent and constrained refinement
Δρ_max_ = 0.73 e Å^−3^
Δρ_min_ = −1.39 e Å^−3^

### 

Data collection: *SMART* (Siemens, 1995 ▶); cell refinement: *SAINT* (Siemens, 1995 ▶); data reduction: *SAINT* and *XPREP* (Siemens, 1995 ▶); program(s) used to solve structure: *SIR97* (Altomare *et al.*, 1999 ▶); program(s) used to refine structure: *SHELXL97* (Sheldrick, 2008 ▶); molecular graphics: *ORTEP-3* (Farrugia, 1997 ▶), *WebLab ViewerPro* (Molecular Simulations, 2000 ▶) and *POV-RAY* (Cason, 2002 ▶).; software used to prepare material for publication: *enCIFer* (Allen *et al.*, 2004 ▶).

## Supplementary Material

Crystal structure: contains datablocks global, I. DOI: 10.1107/S1600536808028365/wm2194sup1.cif

Structure factors: contains datablocks I. DOI: 10.1107/S1600536808028365/wm2194Isup2.hkl

Additional supplementary materials:  crystallographic information; 3D view; checkCIF report

## Figures and Tables

**Table 1:** Selected bond lengths (Å)

Co1—O3	1.897 (3)
Co1—O4	1.941 (3)
Co1—O2	1.992 (3)
Co1—O1	2.002 (3)
Co2—O9	1.887 (3)
Co2—O5	1.949 (3)
Co2—O1	1.986 (3)
Co2—O2^i^	2.054 (3)
Co3—O6	2.019 (3)
Co3—O7	2.061 (3)
Co3—O8	2.065 (3)
Co3—O8^ii^	2.075 (3)
Co3—O5^iii^	2.108 (3)
Co3—O3^iv^	2.416 (3)
P1—O6	1.513 (3)
P1—O4^i^	1.534 (3)
P1—O2^v^	1.560 (3)
P1—O1	1.561 (3)
P2—O9	1.511 (3)
P2—O8^vi^	1.544 (3)
P2—O3^vii^	1.549 (3)
P2—O5^vi^	1.565 (3)

Symmetry codes: (i) 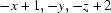; (ii) 
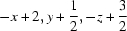
; (iii) 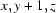; (iv) 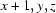; (v) 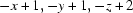; (vi) 
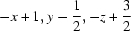
; (vii) 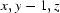.

**Table 2:** Hydrogen-bond geometry (Å, °)

*D*—H⋯*A*	*D*—H	H⋯*A*	*D*⋯*A*	*D*—H⋯*A*
O7—H2⋯O6^iii^	0.90 (3)	1.86 (4)	2.753 (4)	170 (5)
O7—H1⋯O4^viii^	0.903 (10)	1.864 (15)	2.758 (4)	171 (5)

Symmetry codes: (iii) 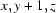; (viii) 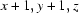.

**Fig. 1.**
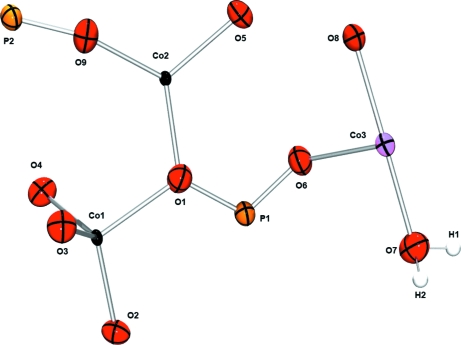
The asymmetric unit of compound (I), drawn with displacement parameters at the 50% probability level. H atoms are given as spheres of arbitrary radius.

**Fig. 2.**
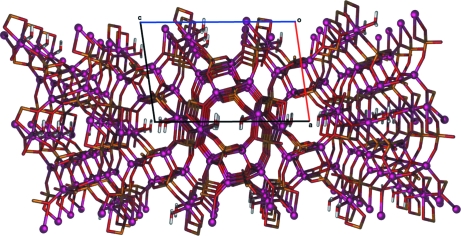
A schematic representation of a section of the three-dimensional network of (I) in a projection along [010]. Hydrogen atoms are omitted for clarity.

## supplementary crystallographic information

### Comment

Synthesis and structural investigations of transition-metal phosphates under various conditions, including high temperature and high pressure, have been investigated for many years (Murugavel *et al.*, 2008). This is not only because of the multifarious structural chemistry, but also due to many potential applications. For a listing of reviews on these materials, see Lee *et al.* (2008). We are currently investigating the synthesis of a variety of similar functional materials through templation effects under hydrothermal conditions. The title compound, Co_3_(PO_4_)_2_.H_2_O, (I), and the related compound Co_3_(PO_4_)_2_.4H_2_O (Lee *et al.*, 2008) were synthesized as a part of these studies.

In the past, many different cobalt(II) orthophosphates have been described, ranging from the anhydrous form Co_3_(PO_4_)_2_ to its corresponding octahydrate (Mellor, 1935). In 1976 Anderson *et al.* reported a first polymorph of Co_3_(PO_4_)_2_.H_2_O formed under high pressure conditions. The second polymorph of Co_3_(PO_4_)_2_.H_2_O presented here has a different unit cell and a considerably different cell volume (638.3 (Anderson *et al.*, 1976) versus 704.1 Å^3^ (this study)) and exhibits also a different assembly of the structural building units. The second polymorph (I) is isotypic with its Zn analogue Zn_3_(PO_4_)_2_.H_2_O (Riou *et al.*, 1986).

The structure of (I) contains three different Co^2+^ centres bridged by orthophosphate anions (Fig 1). The coordination spheres of Co1 and Co2 are distorted tetrahedral while that of Co3 is distorted octahedral, with one considerably longer Co—O bond of 2.416 (3) Å (Table 1). Co1 and Co2 are bonded to the O atoms of four phosphate ligands, whereas Co3 is bonded to five O atoms of four phosphate ligands (one bidentate) and the sixth coordination site is occupied by a water molecule. This assembly leads to the formation of a three-dimensional framework (Fig. 2), which is stabilized by additional O—H···O hydrogen bonds (Table 2).

### Experimental

Conditions of the hydrothermal single crystal growth of the hydrous cobalt(II) orthophosphates Co_3_(PO_4_)_2_.H_2_O and Co_3_(PO_4_)_2_.4 H_2_O  were described in detail in a preceding communication (Lee *et al.*, 2008).

### Refinement

Water H atoms were located in difference Fourier maps and were refined with *U*_iso_(H) values fixed at 1.5*U*_eq_ of the parent O atoms. O—H bond length restraints of 0.89 (1) Å were also employed. The highest peak and the deepest hole in the final Fourier map are located 1.74 Å from O1 and 0.20 Å from P1, respectively.

### Crystal data

**Table:**

Co_3_(PO_4_)_2_·H_2_O	*F*(000) = 740
*M**_r_* = 384.75	*D*_x_ = 3.629 Mg m^−^^3^
Monoclinic, *P*2_1_/*c*	Mo *K*α radiation, λ = 0.71073 Å
Hall symbol: -P 2ybc	Cell parameters from 4684 reflections
*a* = 8.7038 (15) Å	θ = 2.5–28.4°
*b* = 4.8667 (9) Å	µ = 7.47 mm^−^^1^
*c* = 16.705 (3) Å	*T* = 150 K
β = 95.670 (3)°	Plate, purple
*V* = 704.1 (2) Å^3^	0.46 × 0.14 × 0.08 mm
*Z* = 4	

### Data collection

**Table:**

Siemens SMART 1000 CCD diffractometer	1697 independent reflections
Radiation source: sealed tube	1603 reflections with *I* > 2σ(*I*)
graphite	*R*_int_ = 0.026
ω scans	θ_max_ = 28.4°, θ_min_ = 2.4°
Absorption correction: multi-scan (SADABS; Sheldrick, 1999)	*h* = −11→11
*T*_min_ = 0.247, *T*_max_ = 0.554	*k* = −6→6
6569 measured reflections	*l* = −21→21

### Refinement

**Table:**

Refinement on *F*^2^	Primary atom site location: structure-invariant direct methods
Least-squares matrix: full	Secondary atom site location: difference Fourier map
*R*[*F*^2^ > 2σ(*F*^2^)] = 0.033	Hydrogen site location: difference Fourier map
*wR*(*F*^2^) = 0.095	H atoms treated by a mixture of independent and constrained refinement
*S* = 1.07	*w* = 1/[σ^2^(*F*_o_^2^) + (0.0597*P*)^2^ + 3.2629*P*] where *P* = (*F*_o_^2^ + 2*F*_c_^2^)/3
1697 reflections	(Δ/σ)_max_ < 0.001
133 parameters	Δρ_max_ = 0.74 e Å^−^^3^
2 restraints	Δρ_min_ = −1.39 e Å^−^^3^

### Special details

**Table:**

Experimental. The crystal was coated in Exxon Paratone N hydrocarbon oil and mounted on a thin mohair fibre attached to a copper pin. Upon mounting on the diffractometer, the crystal was quenched to 150(K) under a cold nitrogen gas stream supplied by an Oxford Cryosystems Cryostream.
Geometry. All esds (except the esd in the dihedral angle between two l.s. planes) are estimated using the full covariance matrix. The cell esds are taken into account individually in the estimation of esds in distances, angles and torsion angles; correlations between esds in cell parameters are only used when they are defined by crystal symmetry. An approximate (isotropic) treatment of cell esds is used for estimating esds involving l.s. planes.
Refinement. Refinement of *F*^2^ against ALL reflections. The weighted *R*-factor *wR* and goodness of fit *S* are based on *F*^2^, conventional *R*-factors *R* are based on *F*, with *F* set to zero for negative *F*^2^. The threshold expression of *F*^2^ > σ(*F*^2^) is used only for calculating *R*-factors(gt) *etc*. and is not relevant to the choice of reflections for refinement. *R*-factors based on *F*^2^ are statistically about twice as large as those based on *F*, and *R*- factors based on ALL data will be even larger.

### Fractional atomic coordinates and isotropic or equivalent isotropic displacement parameters (Å^2^)

**Table:**

	*x*	*y*	*z*	*U*_iso_*/*U*_eq_	
Co1	0.32855 (5)	0.20270 (9)	0.94291 (3)	0.00253 (14)	
Co2	0.56640 (5)	−0.20252 (9)	0.84576 (3)	0.00303 (15)	
Co3	0.92910 (6)	0.49852 (10)	0.82254 (3)	0.00814 (16)	
P1	0.68190 (11)	0.29320 (19)	0.94510 (6)	0.0086 (2)	
P2	0.23599 (11)	−0.51187 (19)	0.78087 (6)	0.0087 (2)	
O1	0.5357 (3)	0.1481 (6)	0.90286 (17)	0.0114 (5)	
O2	0.3621 (3)	0.3976 (6)	1.04804 (16)	0.0106 (5)	
O3	0.1907 (3)	0.3427 (6)	0.85760 (17)	0.0119 (5)	
O4	0.2836 (3)	−0.1747 (6)	0.96996 (17)	0.0121 (6)	
O5	0.7445 (3)	−0.2368 (6)	0.78428 (17)	0.0116 (5)	
O6	0.8153 (3)	0.2545 (6)	0.89463 (17)	0.0120 (5)	
O7	0.9749 (4)	0.7679 (6)	0.91711 (18)	0.0137 (6)	
O8	0.9060 (3)	0.1703 (6)	0.74391 (17)	0.0108 (5)	
O9	0.3852 (3)	−0.3512 (6)	0.79103 (18)	0.0148 (6)	
H1	1.0769 (18)	0.797 (12)	0.929 (3)	0.022*	
H2	0.933 (6)	0.936 (5)	0.909 (3)	0.022*	

### Atomic displacement parameters (Å^2^)

**Table:**

	*U*^11^	*U*^22^	*U*^33^	*U*^12^	*U*^13^	*U*^23^
Co1	0.0013 (2)	0.0038 (2)	0.0025 (2)	0.00007 (15)	−0.00013 (17)	0.00053 (15)
Co2	0.0006 (2)	0.0045 (3)	0.0040 (2)	−0.00041 (15)	0.00015 (17)	−0.00098 (15)
Co3	0.0077 (3)	0.0083 (3)	0.0088 (3)	−0.00123 (17)	0.00306 (19)	−0.00064 (17)
P1	0.0070 (5)	0.0090 (5)	0.0098 (5)	−0.0004 (3)	0.0014 (3)	−0.0002 (3)
P2	0.0067 (5)	0.0103 (4)	0.0091 (4)	0.0003 (3)	0.0008 (3)	0.0001 (3)
O1	0.0101 (13)	0.0112 (12)	0.0129 (13)	−0.0009 (10)	0.0004 (10)	−0.0005 (10)
O2	0.0114 (13)	0.0106 (13)	0.0099 (13)	0.0026 (10)	0.0011 (10)	−0.0003 (10)
O3	0.0121 (13)	0.0133 (13)	0.0102 (13)	−0.0003 (11)	0.0007 (11)	0.0014 (10)
O4	0.0135 (14)	0.0116 (13)	0.0110 (13)	−0.0005 (10)	−0.0004 (11)	0.0009 (10)
O5	0.0108 (14)	0.0131 (12)	0.0114 (13)	0.0007 (10)	0.0041 (10)	0.0008 (10)
O6	0.0106 (14)	0.0132 (12)	0.0127 (13)	−0.0009 (11)	0.0035 (11)	0.0007 (10)
O7	0.0110 (14)	0.0132 (13)	0.0164 (14)	0.0012 (11)	−0.0014 (11)	−0.0015 (11)
O8	0.0075 (13)	0.0122 (13)	0.0131 (13)	−0.0006 (10)	0.0026 (10)	−0.0022 (10)
O9	0.0109 (14)	0.0163 (13)	0.0171 (14)	−0.0033 (11)	0.0012 (11)	−0.0011 (11)

### Geometric parameters (Å, °)

**Table:**

Co1—O3	1.897 (3)	Co3—O3^iv^	2.416 (3)
Co1—O4	1.941 (3)	Co3—P2^v^	2.8266 (12)
Co1—O2	1.992 (3)	P1—O6	1.513 (3)
Co1—O1	2.002 (3)	P1—O4^i^	1.534 (3)
Co2—O9	1.887 (3)	P1—O2^vi^	1.560 (3)
Co2—O5	1.949 (3)	P1—O1	1.561 (3)
Co2—O1	1.986 (3)	P2—O9	1.511 (3)
Co2—O2^i^	2.054 (3)	P2—O8^vii^	1.544 (3)
Co3—O6	2.019 (3)	P2—O3^viii^	1.549 (3)
Co3—O7	2.061 (3)	P2—O5^vii^	1.565 (3)
Co3—O8	2.065 (3)	P2—Co3^ix^	2.8266 (12)
Co3—O8^ii^	2.075 (3)	O7—H1	0.903 (10)
Co3—O5^iii^	2.108 (3)	O7—H2	0.90 (3)
			
O3—Co1—O4	112.81 (13)	O4^i^—P1—O2^vi^	108.77 (15)
O3—Co1—O2	121.22 (12)	O6—P1—O1	109.15 (16)
O4—Co1—O2	105.11 (12)	O4^i^—P1—O1	108.94 (16)
O3—Co1—O1	108.70 (12)	O2^vi^—P1—O1	105.94 (16)
O4—Co1—O1	99.28 (12)	O9—P2—O8^vii^	112.91 (17)
O2—Co1—O1	107.42 (12)	O9—P2—O3^viii^	115.46 (17)
O9—Co2—O5	112.44 (13)	O8^vii^—P2—O3^viii^	102.81 (16)
O9—Co2—O1	114.62 (13)	O9—P2—O5^vii^	106.80 (16)
O5—Co2—O1	118.58 (12)	O8^vii^—P2—O5^vii^	110.75 (16)
O9—Co2—O2^i^	114.12 (12)	O3^viii^—P2—O5^vii^	108.04 (16)
O5—Co2—O2^i^	103.10 (12)	O9—P2—Co3^ix^	141.79 (13)
O1—Co2—O2^i^	91.48 (11)	O8^vii^—P2—Co3^ix^	45.96 (10)
O6—Co3—O7	89.20 (12)	O3^viii^—P2—Co3^ix^	58.69 (11)
O6—Co3—O8	84.37 (11)	O5^vii^—P2—Co3^ix^	110.73 (12)
O7—Co3—O8	168.15 (12)	P1—O1—Co2	117.61 (16)
O6—Co3—O8^ii^	164.15 (12)	P1—O1—Co1	120.63 (16)
O7—Co3—O8^ii^	93.56 (12)	Co2—O1—Co1	116.29 (14)
O8—Co3—O8^ii^	90.02 (7)	P1^vi^—O2—Co1	120.56 (16)
O6—Co3—O5^iii^	97.82 (11)	P1^vi^—O2—Co2^i^	115.98 (15)
O7—Co3—O5^iii^	85.85 (12)	Co1—O2—Co2^i^	123.17 (15)
O8—Co3—O5^iii^	104.85 (11)	P2^iii^—O3—Co1	126.29 (18)
O8^ii^—Co3—O5^iii^	97.95 (11)	P1^i^—O4—Co1	123.07 (17)
O6—Co3—O3^iv^	100.18 (11)	P2^x^—O5—Co2	116.94 (17)
O7—Co3—O3^iv^	84.70 (11)	P2^x^—O5—Co3^viii^	120.43 (16)
O8—Co3—O3^iv^	86.65 (10)	Co2—O5—Co3^viii^	120.96 (14)
O8^ii^—Co3—O3^iv^	64.61 (10)	P1—O6—Co3	135.08 (18)
O5^iii^—Co3—O3^iv^	159.51 (11)	Co3—O7—H1	113 (4)
O6—Co3—P2^v^	131.88 (9)	Co3—O7—H2	115 (4)
O7—Co3—P2^v^	94.83 (9)	H1—O7—H2	105 (5)
O8—Co3—P2^v^	82.21 (8)	P2^x^—O8—Co3	129.77 (16)
O8^ii^—Co3—P2^v^	32.34 (8)	P2^x^—O8—Co3^xi^	101.70 (14)
O5^iii^—Co3—P2^v^	130.28 (8)	Co3—O8—Co3^xi^	128.53 (14)
O6—P1—O4^i^	112.20 (17)	P2—O9—Co2	157.0 (2)
O6—P1—O2^vi^	111.63 (16)		
			
O6—P1—O1—Co2	36.4 (2)	O1—Co2—O5—P2^x^	−54.5 (2)
O4^i^—P1—O1—Co2	−86.41 (19)	O2^i^—Co2—O5—P2^x^	−153.51 (18)
O2^vi^—P1—O1—Co2	156.73 (16)	O9—Co2—O5—Co3^viii^	−111.58 (17)
O6—P1—O1—Co1	−170.63 (17)	O1—Co2—O5—Co3^viii^	110.83 (17)
O4^i^—P1—O1—Co1	66.5 (2)	O2^i^—Co2—O5—Co3^viii^	11.79 (18)
O2^vi^—P1—O1—Co1	−50.3 (2)	O4^i^—P1—O6—Co3	−132.6 (2)
O9—Co2—O1—P1	−176.28 (16)	O2^vi^—P1—O6—Co3	−10.2 (3)
O5—Co2—O1—P1	−39.6 (2)	O1—P1—O6—Co3	106.6 (3)
O2^i^—Co2—O1—P1	66.23 (18)	O7—Co3—O6—P1	55.9 (3)
O9—Co2—O1—Co1	29.6 (2)	O8—Co3—O6—P1	−134.0 (3)
O5—Co2—O1—Co1	166.29 (13)	O8^ii^—Co3—O6—P1	156.2 (3)
O2^i^—Co2—O1—Co1	−87.91 (15)	O5^iii^—Co3—O6—P1	−29.8 (3)
O3—Co1—O1—P1	122.72 (19)	P2^v^—Co3—O6—P1	151.72 (19)
O4—Co1—O1—P1	−119.25 (19)	O6—Co3—O8—P2^x^	44.9 (2)
O2—Co1—O1—P1	−10.1 (2)	O7—Co3—O8—P2^x^	102.3 (6)
O3—Co1—O1—Co2	−83.97 (17)	O8^ii^—Co3—O8—P2^x^	−150.0 (2)
O4—Co1—O1—Co2	34.06 (16)	O5^iii^—Co3—O8—P2^x^	−51.7 (2)
O2—Co1—O1—Co2	143.21 (14)	P2^v^—Co3—O8—P2^x^	178.6 (2)
O3—Co1—O2—P1^vi^	−18.9 (2)	O6—Co3—O8—Co3^xi^	−134.86 (19)
O4—Co1—O2—P1^vi^	−148.23 (18)	O7—Co3—O8—Co3^xi^	−77.4 (6)
O1—Co1—O2—P1^vi^	106.71 (19)	O8^ii^—Co3—O8—Co3^xi^	30.29 (16)
O3—Co1—O2—Co2^i^	154.62 (15)	O5^iii^—Co3—O8—Co3^xi^	128.52 (17)
O4—Co1—O2—Co2^i^	25.34 (19)	P2^v^—Co3—O8—Co3^xi^	−1.19 (16)
O1—Co1—O2—Co2^i^	−79.72 (18)	O8^vii^—P2—O9—Co2	116.3 (5)
O4—Co1—O3—P2^iii^	−129.2 (2)	O3^viii^—P2—O9—Co2	−1.5 (6)
O2—Co1—O3—P2^iii^	105.0 (2)	O5^vii^—P2—O9—Co2	−121.7 (5)
O1—Co1—O3—P2^iii^	−20.1 (2)	Co3^ix^—P2—O9—Co2	69.4 (6)
O3—Co1—O4—P1^i^	−150.21 (19)	O5—Co2—O9—P2	137.1 (5)
O2—Co1—O4—P1^i^	−16.1 (2)	O1—Co2—O9—P2	−83.5 (6)
O1—Co1—O4—P1^i^	94.9 (2)	O2^i^—Co2—O9—P2	20.1 (6)
O9—Co2—O5—P2^x^	83.1 (2)		

Symmetry codes: (i) −*x*+1, −*y*, −*z*+2; (ii) −*x*+2, *y*+1/2, −*z*+3/2; (iii) *x*, *y*+1, *z*; (iv) *x*+1, *y*, *z*; (v) *x*+1, *y*+1, *z*; (vi) −*x*+1, −*y*+1, −*z*+2; (vii) −*x*+1, *y*−1/2, −*z*+3/2; (viii) *x*, *y*−1, *z*; (ix) *x*−1, *y*−1, *z*; (x) −*x*+1, *y*+1/2, −*z*+3/2; (xi) −*x*+2, *y*−1/2, −*z*+3/2.

### Hydrogen-bond geometry (Å, °)

**Table:**

*D*—H···*A*	*D*—H	H···*A*	*D*···*A*	*D*—H···*A*
O7—H2···O6^iii^	0.90 (3)	1.86 (4)	2.753 (4)	170 (5)
O7—H1···O4^v^	0.90 (1)	1.86 (2)	2.758 (4)	171 (5)

Symmetry codes: (iii) *x*, *y*+1, *z*; (v) *x*+1, *y*+1, *z*.
